# NU-IN: Nucleotide evolution and input module for the EvolSimulator genome simulation platform

**DOI:** 10.1186/1756-0500-3-217

**Published:** 2010-08-02

**Authors:** Katrina M Dlugosch, Michael S Barker, Loren H Rieseberg

**Affiliations:** 1 Department of Botany, University of British Columbia, Vancouver, BC V6T1Z4, Canada; 2 The Biodiversity Research Centre, University of British Columbia, Vancouver, BC V6T1Z4, Canada; 3 Department of Biology and Center for Genomics and Bioinformatics, Indiana University, Bloomington, IN 47405, USA

## Abstract

**Background:**

There is increasing demand to test hypotheses that contrast the evolution of genes and gene families among genomes, using simulations that work across these levels of organization. The EvolSimulator program was developed recently to provide a highly flexible platform for forward simulations of amino acid evolution in multiple related lineages of haploid genomes, permitting copy number variation and lateral gene transfer. Synonymous nucleotide evolution is not currently supported, however, and would be highly advantageous for comparisons to full genome, transcriptome, and single nucleotide polymorphism (SNP) datasets. In addition, EvolSimulator creates new genomes for each simulation, and does not allow the input of user-specified sequences and gene family information, limiting the incorporation of further biological realism and/or user manipulations of the data.

**Findings:**

We present modified C++ source code for the EvolSimulator platform, which we provide as the extension module NU-IN. With NU-IN, synonymous and non-synonymous nucleotide evolution is fully implemented, and the user has the ability to use real or previously-simulated sequence data to initiate a simulation of one or more lineages. Gene family membership can be optionally specified, as well as gene retention probabilities that model biased gene retention. We provide PERL scripts to assist the user in deriving this information from previous simulations. We demonstrate the features of NU-IN by simulating genome duplication (polyploidy) in the presence of ongoing copy number variation in an evolving lineage. This example is initiated with real genomic data, and produces output that we analyse directly with existing bioinformatic pipelines.

**Conclusions:**

The NU-IN extension module is a publicly available open source software (GNU GPLv3 license) extension to EvolSimulator. With the NU-IN module, users are now able to simulate both drift and selection at the nucleotide, amino acid, copy number, and gene family levels across sets of related genomes, for user-specified starting sequences and associated parameters. These features can be used to generate simulated genomic datasets under an extremely broad array of conditions, and with a high degree of biological realism.

## Introduction

The current explosion of genomic sequence data is generating unprecedented insights into the structure and evolution of genomes. Among the most profound recent discoveries is the extent to which gene copy number variation and the gain and loss of lineage specific duplications are pervasive and ongoing features of evolution in many organisms [1]. Such paralogs are known to affect phenotypes directly and to play important roles in the evolution of gene functions and divergence among species, e.g. [2-4]. Accordingly, their retention or loss appears to be shaped by selection in some cases [5]. This means that comprehensive studies of genomes must incorporate evolution at the levels of gene and gene family loss and gain, as well as the traditional scales of nucleotide and amino acid mutation. Simulations that integrate across all of these levels to generate different evolutionary scenarios will provide powerful tools for testing hypotheses about how evolution works.

The simulation platform EvolSimulator [6] was recently developed to accommodate many of these needs. It generates the coding sequences of an ancestral haploid genome, uses this genome to initiate any number of lineages, and evolves the lineages forward under different selective regimes. Users can specify rates of nucleotide mutation, gene duplication and loss, as well as lateral gene transfer, if desired. EvolSimulator is currently one of the only programs available for performing such detailed genome-scale simulations, and it provides a particularly powerful platform for testing aspects of gene family evolution [7].

Currently, EvolSimulator (as of version 2.1.0) is designed to simulate only amino acid evolution. A nucleotide sequence exists for every genome, and mutations are evaluated at each position according to nucleotide substitution models set by the user, but only non-synonymous changes are retained and propagated through the simulation. A full implementation of (synonymous and non-synonymous) nucleotide evolution in the simulation would be highly advantageous for testing hypotheses at the nucleotide level, and for comparison to increasingly available genome, transcriptome, and single nucleotide polymorphism (SNP) datasets. Many analyses of sequence and genome evolution utilize patterns of both synonymous and non-synonymous nucleotide changes to infer relationships within and between genomes and distinguish the action of selection [8]. Simulations that include neutral and non-neutral nucleotide evolution are therefore particularly important for evolutionary hypothesis testing.

In addition, particular evolutionary scenarios will often require specific data types, different parameter sets at different time points, and/or user manipulations of the data. These objectives are most easily achieved by allowing users to input their own sequence and other genomic information as the starting material for a given simulation. For example, it is desirable to use data from real organisms in cases where the simulation will be compared directly to real data, or used to test analysis pipelines which are dependent on biological realism (such as protein motifs). It is also commonly helpful to restart a simulation, for instance to provide replication from the same starting genome, multi-stage simulations where different parameter combinations are needed, and manual manipulation of genomes (such as whole genome duplication, see example below) within the simulation. EvolSimulator is not designed to accommodate these inputs at present, and instead generates a new genome as the ancestor for each simulation. We have developed NU-IN, an extension to the EvolSimulator platform to implement nucleotide evolution and offer user data input, along with the existing program features (see Additional File 1: NU-IN Download 1.1.0 for the source code and documentation for this software).

### Software Description

NU-IN takes advantage of EvolSimulator's existing nucleotide-based mutation machinery and implements it fully. EvolSimulator generates nucleotide changes according to user specified rates of mutation and nucleotide bias. Previously, only mutations leading to an amino acid change that survived selection were recorded. With NU-IN, all synonymous (silent) nucleotide mutations as well as the non-synonymous mutations are reported. Synonymous changes are selectively neutral and remain in the genome unless they co-occur with an amino acid change that is eliminated by selection (as we imagine happens in nature).

In addition, NU-IN allows users to start simulations with their own data, including genes from real organisms and/or genomes already generated by EvolSimulator. Input genomes may be any fasta-formatted nucleotide sequences in reading frame. NU-IN processes input data in several steps: First, the genome file is read, the genes are numbered sequentially, and each gene is assigned as the first member of its gene family. Every gene is then assigned a 'usefulness' value in all selective environments ('habitats' and 'niches') to be used in the program. Usefulness values are probabilities of gene retention, dictating how important it is to prevent a gene from being lost in an environment if the genome size happens to shrink (where parameters for genome size variation are set by the user). This represents selection at the level of gene loss. All of these steps mimic the same process used by EvolSimulator2.1.0 when it creates a simulated starting genome.

NU-IN also creates the option for the user to provide their own gene family membership and gene usefulness information. When inputting real data, gene family membership information can be derived from additional analyses of gene relationships in the genome [9]. Gene usefulness could be tied to knowledge about historical patterns of gene retention (such as biased retention of transcription factors [10]). For inputs of genomes generated by previous runs of the simulation, gene family and usefulness information is embedded in the output files. The NU-IN download provides several PERL scripts to aid users in parsing sequence, family, and usefulness information from the simulation output.

### Example Usage

**Figure 1:**
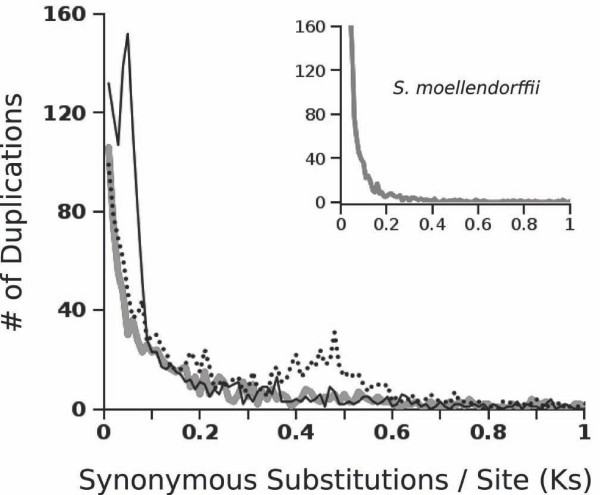
**Simulated Ks Distributions of Duplicated Genes**. Frequency distributions of synonymous site divergence (Ks) at nodes of gene family trees for loci in a sample of the *S. moellendorffii *genome (inset), a simulated genome based on those same loci (grey line), and a whole genome duplication of the simulated genes that has since evolved for 0.05 Ks (solid line) and 0.45 Ks (dashed line).

We expect that users will find this to be an exceptionally practical tool with which they can test a very broad array of hypotheses about sequence and genome evolution. For example, simulated datasets can be generated to evaluate the impact of mutation rate, divergence times, and/or selection on our ability to detect reticulate evolution (lateral gene transfer or hybridization), copy number variation, or genome duplication in samples of genomic data. To demonstrate one such an approach, we simulated a whole genome duplication (polyploidy) in an evolving lineage with a constant background rate of gene gain and loss. We initiated our simulation with coding sequences from the spike moss *Selaginella moellendorffii *genome (10 Sep 2008 release [11]), which shows no evidence of recent or ancient polyploidy. We evolved this genome for 5.0 Ks (synonymous substitutions/site) to allow it to stabilize on a constant rate of gene turnover (see Additional File 2: Example Parameters for the parameters used), and produce an age distribution of simulated paralogs that is similar to the original data (Figure 1 inset). Using the output of this simulation, we doubled the genome manually and input this polyploid genome and its gene family and usefulness information back into the simulation to evolve for an additional 0.05 and 0.45 Ks.

For the original, simulated, and duplicated genomes, we used an existing bioinformatic pipeline to identify the Ks values for each duplication event in each gene family tree [12,13] (available as 'DupPipe' at EvoPipes.net [14]). We used a random sample of 10,000 genes for these analyses, similar to what is available in many publicly available EST datasets (e.g. [12]). Briefly, this pipeline identifies sets of similar sequences as gene family members, uses alignment with known plant proteins to place sequences in reading frame, generates a gene tree for the family, and calculates the synonymous site divergence that corresponds to each node (duplication event) in the tree. Because our simulations were based on sequences from a real genome, we were able to use the existing analysis pipeline unaltered, and produce observations with the same error (e.g., in defining gene families and assigning reading frame) as occurs with real data.

Distributions of the Ks values for each duplication event are commonly used to evaluate evidence for ancient genome duplications, apparent as peaks of duplication at particular levels of divergence (a proxy for time) [15]. Detailed simulations of the sequences that yield these distributions have not been available previously, however, limiting tests of the factors generating and shaping these patterns. Histograms of duplication Ks values for our simulations (Figure 1) demonstrate that the program achieved realistic patterns of gene gain and loss, and that whole genome duplications are clearly visible as peaks that diminish with time since polyploidization. The reduction in peak prominence is due to ongoing paralog loss, and such simulations will be invaluable for generating expectations about how far back in time these events might remain observable by this method.

## Conclusions

Genomic datasets offer tremendous potential to address broad evolutionary questions, but demand analytical tools that work at these same scales of biological organization. NU-IN expands the EvolSimulator platform to accommodate hypotheses involving synonymous and non-synonymous nucleotide evolution, and allows users to provide and manipulate input data as needed to address their unique needs. These features can be used to generate simulated genomic datasets under an extremely broad array of conditions affecting point mutations, copy number variation, lateral gene transfer, drift, and selection at multiple levels. Our simulation of a genome duplication event demonstrates the ability of this platform to produce realistic genome-wide patterns of gene divergence and variation from these fundamental evolutionary processes.

## Availability

**• Project name**: NU-IN

**• Project home page**: http://evopipes.net/nuin.html

**• Operating system(s)**: Linux/Unix (gcc/g++ compiler)

**• Programming language**: C++, PERL

**• License**: GNU GPL

## Competing interests

The authors declare that they have no competing interests.

## Authors' contributions

KMD, MSB, and LHR conceived of the software development. KMD wrote the software and manuscript. All authors read and approved the final manuscript.

## Supplementary Material

Additional file 1 **Example Parameters**. A parameter text file used to run the NU-IN simulation program. Click here for file

Additional file 2 **NUIN Download 1.0.2**. An archive folder (gzip tarball) of documentation and source code files for NU-IN version 1.0.2. Click here for file
