# Supplementary material for: NU-IN: Nucleotide evolution and input module for the EvolSimulator genome simulation platform
Source: BMC Res Notes. 2010 Aug 2;3:217. doi: 10.1186/1756-0500-3-217 (PMC3161368; doi:10.1186/1756-0500-3-217)
Supplement: Additional file 1 — Example Parameters. A parameter text file used to run the NU-IN simulation program. [file 1756-0500-3-217-S1.TGZ › NuIn_1.0.2/NUIN_Manual_1.0.2.pdf]

NU-IN
Nucleotide Evolution and Input Module for EvolSimulator2.1.0
version 1.0.2 Manual

CONTENTS

1. What's new in NU-IN 1
    1.1 A brief overview of EvolSimulator2.1.0 1
    1.2 Features added by NU-IN 2
        1.2.1 Nucleotide evolution
        1.2.2 Input of genome sequences and related information
    1.3 Changes to existing parameters in EvolSimulator2.1.0 3

2. Installation 4

3. Usage 4
    3.1 Preparing the parameter and input files 4
    3.2 Preparing EvolSimulator output for input into a new simulation 6
        3.2.1 Creating a genome input file from the *.gbk EvolSimulator output
        3.2.2 Gathering gene family information from your genome file
        3.2.3 Gathering gene usefulness information for your genome file

4. Citing NU-IN 7

5. License and Disclaimer 7

1. WHAT'S NEW IN NU-IN

1.1 Overview of EvolSimulator2.1.0

EvolSimulator (Beiko & Charlebois 2007 Bioinformatics 23:825-831) is a simulation platform for the coding regions of genomes. The user tells the program how many genes of what size and amino acid composition to create. A haploid genome is created (both the amino acid and the underlying nucleotide sequences), and then it is allowed to evolve in amino acid sequence and in the number of genes in the genome. 'Speciation' events are allowed as set by the user, and so phylogenies can be generated. The various evolving lineages can occupy different 'habitats' and 'niches', which then each impose selection for evolution toward different randomly generated optima for a subset of the genes in the genome. These environments also dictate which loci and gene families are most 'useful' in that habitat/niche, and these are prevented from being lost as genome size fluctuates. Lateral gene transfer is also possible. The many options provided for shaping all of these

events make for a very flexible simulation program for lineages of coding sequences evolving under selection, copy number variation, and/or gene transfer.

1.2 Features added by NU-IN

NU-IN was designed to expand EvolSimulator in two fundamental ways: 1) Allow non-synonymous nucleotide evolution and 2) Permit input of your own genome sequences and related information:

1.2.1 *Nucleotide evolution*

In its original implementation, EvolSimulator is designed to model only amino acid evolution. It does keep a record of the underlying nucleotide sequence, but when it evaluates mutations (both non-synymous and synonymous) for each codon, it updates the nucleotide sequence only if an amino acid change survives selection. Essentially, silent mutations are ignored and not recorded. Confusingly, some silent mutations can be retained and observed if they happen to co-occur with a non-synonymous mutation at the same step in the simulation.

NU-IN changes the behavior of the simulation to report all synonymous changes. These are selectively neutral and remain in the genome unless they co-occur with an amino acid change that is eliminated by selection (as we imagine happens in nature). This change expands EvolSimulator2.1.0 from being exclusively an amino acid sequence simulation program to being a nucleotide sequence simulation program as well. Among many possibilities, the simulated data are now appropriate for tests of selection and estimates of divergence times based on synonymous site divergence.

1.2.2 *Input of genomes, gene family membership, and gene 'usefulness'*

EvolSimulator2.1.0 functions by generating a simulated genome as the common ancestor of all lineages created in the simulation. NU-IN expands this to offer users the means to start simulations with genomes (coding sequences) from real organisms or to take genomes already generated by EvolSimulator simulations and continue their evolution in future simulations. It is desirable to use data from real organisms in cases where the simulation will be compared directly to real data, or used to test analysis pipelines which are dependent on biological realism (such as protein motifs). It is also commonly helpful to restart a simulation for any number of reasons (e.g. replication from the same starting genome, multi-stage simulations where different parameter combinations are needed, and manual manipulation of genomes such as whole genome duplication within the simulation).

NU-IN accomplishes this in several steps: First, the genome (provided as a fasta file in reading frame) is obtained, the genes are numbered sequentially, and each gene is assigned as the first member of its gene family. Every gene is then assigned a 'usefulness' value in all habitats and niches to be used in the program. Usefulness values are probabilities of gene retention, dictating how important it is to prevent a gene from being lost in that environment if the genome size happens to shrink (parameters for genome size variation are set by the user). All of these steps mimic the same process used by EvolSimulator2.1.0 when it creates

a starting genome.

NU-IN also creates the option for the user to provide their own gene family membership and gene usefulness information. This is straightforward and recommended when using output from a previous simulation; the information is provided in the output (see Section 3.2 below). When inputing real data, gene family membership information will have to come from previous analyses of gene similarity/relationships in the genome. Gene usefulness information is optional, but might be desirable in some cases, e.g. in studies of biased gene retention.

Some features of gene family evolution have been modified from EvolSimulator2.1.0 in order to make these additions possible/practical and more biologically realistic; these changes are described below in Section 1.3.

1.3 Changes to existing parameters in EvolSimulator2.1.0

Several existing parameters function differently under the NU-IN module (please see the EvolSimulator2.1.0 manual for complete original descriptions):

`inertiaDriftFactor`

Null in NU-IN. Inertia was the tendency of a gene to resist mutation of any kind, effectively generating loci with overall differences in mutation rate. This inertia has been eliminated in NU-IN and resistance to mutation is only governed by selection on amino acid changes (which remains locus-specific). This change was made to allow repeated simulations to be comparable, without having to transfer locus-specific inertia information (as well as selection strength information) across simulations.

`maxInitialSelectivePressure`

Defines the *initial* selective pressure on a new gene in NU-IN (as a constant, no longer a maximum). This applies to both genes input or created at the start of the simulation, and genes created later by duplication. In NU-IN, selection on a gene can still change over time, as governed by the selectiveDrift parameters. This change aids carryover of selective constraint on loci across runs, and also allows much more control in general over selection on individual loci, since this value is no longer drawn randomly from a distribution.

`Prop*Req parameters` `(*global, habitat, and niche)`

This defines the proportions of gene families strictly required in particular environments (last member cannot be lost). These are null in NU-IN *if* gene family information is given as an input file. In that case, the last member of a gene family can never be lost in any lineage, for all gene families (fixed because total family loss happens very rarely in nature). If no family information is input, then all ancestral genes initiate their own families, but these will vary in the degree to which they can be lost, as described by the Prop*Req parameters.

`duplicabilityDriftFactor`

Null in NU-IN. Duplicability was the propensity of a gene to duplicate. In NU-IN, all loci

have an equal probability of duplicating (though note that big gene families will still tend to get bigger, because by chance their members will be selected more often for random duplication). This change was made to allow repeated simulations to be more comparable, without having to transfer family-specific duplicability information across simulations.

TransitionProb parameters (min, max, and starting values)

Transition probabilities previously included mutation to the same nucleotide (effectively no mutation), and this has been changed such that transitions are always nucleotide changes.

numProteinTypes

This should be set to zero ('0') in order to take in an input genome fasta file. This was a cryptic switch for internal testing in EvolSimulator 2.1.0, now more fully implemented in NU-IN.

2. INSTALLATION

NU-IN is is an extension module for the EvolSimulator 2.1.0 program, which must be downloaded first (http://bioinformatics.org.au/evolsim/). Modified versions of six of the EvolSimulator source code files are provided in the src folder in the NU-IN download. To implement NU-IN, simply copy the new source code files over the old and compile EvoSimulator as directed in its documentation.

NU-IN also provides some additional scripts in the parsing_scripts folder (see 'USAGE' section). These are written in PERL (on a Linux platform) and require that PERL be installed.

3. USAGE

3.1 Preparing the parameter and input files

NU-IN requires a modified parameter file, a examples of which ('NuIn_Params_*_Example.txt') are provided in the NU-IN download. These include three parameters new to EvolSimulator 2.1.0, explained below:

- fileName_of_gene_DNA_sequences_in_FASTA_format
- filename_of_family_IDs_for_each_sequence
- filename_of_usefulness_values_for_each_sequence

fileName_of_gene_DNA_sequences_in_FASTA_format

Here a fasta file can be read into the program to function as the starting point for the simulation (the 'cenancestor').

All sequences should:

- Be in reading frame (multiples of 3 bases)
- Include only letters A/a/C/c/G/g/T/t
- Have lengths within the bounds set (by user) for gene lengths in the simulation

- Total up to a genome size that is within the bounds set for genomes in the simulation

Note that the original input names will be lost and the loci renumbered sequentially starting from '1'. If no file will be provided, this line should read 'none'. An example file '002_100.fasta' can be found in the ParsingScripts/ExampleData directory.

`filename_of_family_IDs_for_each_sequence`

Here the user may indicate the filename for a file of gene family information, located in the same directory as the EvolSimulator executable. This will cause the simulation to prevent the loss of the last member of every gene family in a genome during its evolution. Families should be identified with the number of one of the genes in the family (as they appear sequentially in the genome file, starting with '1'), and the file should be a simple list with each line the family for each gene, in the same order as the genome input file (if used). An example file '002_100.families' can be found in the ExampleData directory inside the ParsingScripts directory. If no file will be provided, this line in the parameter file should read 'none', and the simulation will assign every ancestral gene to its own family.

Example:

My input genome file has four genes and looks like...

```
>geneW
ACGT...
>geneX
ACGT...
>geneY
ACGT...
>geneZ
ACGT...
```

I want to indicate that geneX and geneZ are in the same family; my family file will look like...

```
1
2
3
2
```

`filename_of_usefulness_values_for_each_sequence`

Here the user may indicate the filename for a file of gene usefulness information (see EvolSimulator parameter 'paralogUsefulnessFactor' for description), located in the same directory as the EvolSimulator executable. If no file will be provided, this line should read 'none'.

For information on how to create a usefulness file from output of a previous simulation, refer to Section 3.2 below.

To create a usefulness file from scratch, provide usefulness values for each gene on a

separate line, in the same order as the input genome. These values are probabilities of being retained, so they should be between 0.0 and 1.0, where '1.0' indicates that the gene will never be lost. Each line should have four values, separated by SPACES:

- Integer index for the gene
- Global gene usefulness
- Habitat gene usefulness
- Niche gene usefulness

Example:
For my four genes (W,X,Y,Z) in the example above, the file might look like...

```
1 0.58 0.03 0.48
2 0.01 0.23 0.50
3 0.58 0.56 0.14
4 0.01 0.09 0.58
```

3.2 Preparing EvolSimulator output for input into a new simulation

3.2.1 *Creating a genome input file from the *.gbk EvolSimulator output*

EvolSimulator/NU-IN outputs genomes as Genbank files (*.gbk). To convert gbk format to fasta format for input, the program gbk_to_fasta.pl is provided in the ParsingScripts directory of the NU-IN download. To use run:

perl gbk_to_fasta.pl
-i: <*.gbk> input [specify directory if appropriate, e.g. "-i ExampleData/002_100.gbk"]
-o: <output_filename> [specify directory if desired, e.g. "-o ExampleData/002_100.fasta"]

3.2.2 *Gathering gene family information from your genome file*

Gene family files are lists of family IDs, where the family ID is the index of one member of the family as it appears in the input genome (see parameter file description in Section 3.1 above for more detail). NOTE: If you intend to modify the list of genes in your genome input file (deleting or duplicating genes), do so before you create the family file!

The program get_families.pl is provided in the ParsingScripts directory of the NU-IN download. To use run:

perl get_families.pl
-i: <fasta_file> input [specify directory if appropriate, e.g. "-i ExampleData/002_100.fasta"]
-o: <output_filename> [specify directory if desired, e.g. "-o ExampleData/002_100.families"]

3.2.3 *Gathering gene usefulness information for your genome file*

EvolSimulator/NU-IN will generate a geneCounts.txt file at the end of any simulation, and this contains the gene usefulness information. All loci have usefulness values for every habitat and niche in the simulation, regardless of whether they are present in genomes

inhabiting those niches. You can see what habitat and niche a particular genome occupies in the header of its *.gbk output file.

You must choose which habitat and niche you which to draw usefulness values from. When input to the simulation, these values will be propagated to every habitat and niche that is created, i.e. the values for your ancestral genes will not vary among habitats and niches. More detail on the format of usefulness input files can be found in Section 3.1 above.

NOTE: EvolSimulator/NU-IN can output genomes periodically throughout a simulation, but to restart a simulation with gene usefulness information, your genome file must be a set of sequences from one or more genomes present at the END of a simulation, with their locus names intact (duplicating or deleting genes from that output are acceptable modifications). These will have matching information in the geneCounts.txt file.

The program get_usefulness.pl is provided in the ParsingScripts directory of the NU-IN download. To use run:

```
perl get_usefulness.pl
    -i: <fasta_file> genome input [can specify directory, e.g. "-i ExampleData/002_100.fasta"]
    -u: <geneCounts.txt> input file [can specify directory, e.g. "-u ExampleData/geneCounts.txt"]
    -h: <habitat_number> habitat from which to draw habitat usefulness values [default = 0]
    -n: <niche_number> niche within the given habitat for usefulness values [default = 0]
    -o: <output_filename> [can specify directory, e.g. "-o ExampleData/002_100.usefulness"]
```

4. CITING NU-IN

Please cite BOTH the NU-IN module and EvolSimulator2.1.0!!

Citation for NU-IN module:

Dlugosch KM, MS Barker, and LH Rieseberg. NU-IN: Nucleotide evolution and input module for the EvolSimulator genome simulation platform. BMC Research Notes, *In Press* (2010).
Available at: http://www.kdlugosch.net/software/

Citation for EvolSimulator2.1.0:

Beiko RG and RL Charlebois (2007) A simulation test bed for hypotheses of genome evolution. Bioinformatics 23:825-831.
Available at: http://bioinformatics.org.au/evolsim/

5. LICENSE AND DISCLAIMER

NU-IN: Nucleotide Evolution and Input Module for EvolSimulator2.1.0
Copyright (C) 2010 Katrina M Dlugosch

This program is free software: you can redistribute it and/or modify it under the terms of the GNU General Public License as published by the Free Software Foundation, either version 3 of the License, or (at your option) any later version.

This program is distributed in the hope that it will be useful, but WITHOUT ANY WARRANTY; without even the implied warranty of MERCHANTABILITY or FITNESS FOR A PARTICULAR PURPOSE. See the GNU General Public License for more details, available as License.txt in your NU-IN download, and at <http://www.gnu.org/licenses/>.

At this time, this program is distributed without any guarantee of technical support. For contact information and regular updates, see the NU-IN website <http://www.kdlugosch.net/software/>.
